# Simultaneous Tc-99m and I-123 dual-radionuclide imaging with a solid-state detector-based brain-SPECT system and energy-based scatter correction

**DOI:** 10.1186/s40658-016-0147-2

**Published:** 2016-06-29

**Authors:** Wataru Takeuchi, Atsuro Suzuki, Tohru Shiga, Naoki Kubo, Yuichi Morimoto, Yuichiro Ueno, Keiji Kobashi, Kikuo Umegaki, Nagara Tamaki

**Affiliations:** Research and Development Group, Hitachi Ltd, 1-280, Higashi-Koigakubo, Kokubunji-shi, 185-8601 Tokyo Japan; Department of Nuclear Medicine, School of Medicine, Hokkaido University, Sapporo, Hokkaido Japan; Office of Health and Safety, Hokkaido University, Sapporo, Hokkaido Japan; Division of Quantum Science and Engineering, Graduate School of Engineering, Hokkaido University, Sapporo, Japan

**Keywords:** Dual radionuclide, Solid-state detector, CdTe, Scatter correction, Crosstalk

## Abstract

**Background:**

A brain single-photon emission computed tomography (SPECT) system using cadmium telluride (CdTe) solid-state detectors was previously developed. This CdTe-SPECT system is suitable for simultaneous dual-radionuclide imaging due to its fine energy resolution (6.6 %). However, the problems of down-scatter and low-energy tail due to the spectral characteristics of a pixelated solid-state detector should be addressed. The objective of this work was to develop a system for simultaneous Tc-99m and I-123 brain studies and evaluate its accuracy.

**Methods:**

A scatter correction method using five energy windows (FiveEWs) was developed. The windows are Tc-lower, Tc-main, shared sub-window of Tc-upper and I-lower, I-main, and I-upper. This FiveEW method uses pre-measured responses for primary gamma rays from each radionuclide to compensate for the overestimation of scatter by the triple-energy window method that is used. Two phantom experiments and a healthy volunteer experiment were conducted using the CdTe-SPECT system. A cylindrical phantom and a six-compartment phantom with five different mixtures of Tc-99m and I-123 and a cold one were scanned. The quantitative accuracy was evaluated using 18 regions of interest for each phantom. In the volunteer study, five healthy volunteers were injected with Tc-99m human serum albumin diethylene triamine pentaacetic acid (HSA-D) and scanned (single acquisition). They were then injected with I-123 *N*-isopropyl-4-iodoamphetamine hydrochloride (IMP) and scanned again (dual acquisition). The counts of the Tc-99m images for the single and dual acquisitions were compared.

**Results:**

In the cylindrical phantom experiments, the percentage difference (PD) between the single and dual acquisitions was 5.7 ± 4.0 % (mean ± standard deviation). In the six-compartment phantom experiment, the PDs between measured and injected activity for Tc-99m and I-123 were 14.4 ± 11.0 and 2.3 ± 1.8 %, respectively. In the volunteer study, the PD between the single and dual acquisitions was 4.5 ± 3.4 %.

**Conclusions:**

This CdTe-SPECT system using the FiveEW method can provide accurate simultaneous dual-radionuclide imaging. A solid-state detector SPECT system using the FiveEW method will permit quantitative simultaneous Tc-99m and I-123 study to become clinically applicable.

## Background

The feasibility of simultaneous multi-radionuclide imaging is a promising advantage of the single-photon emission computed tomography (SPECT) system. The scanning of multi-radionuclide pharmaceuticals will enable various kinds of functional images to be obtained without position error or time difference. Analysis of the relationships among several kinds of functions will contribute to the diagnosis and identification of the mechanisms of various cranial nerve diseases, for example, a set of cerebral perfusion [1] and dopamine transporter [2] imaging is useful for dementia patients to distinguish between Alzheimer’s disease and Lewy body dementia and a set of cerebral perfusion and benzodiazepine receptor imaging [3] is useful to localize the epileptogenic foci.

A number of simultaneous multi-radionuclide imaging studies have been reported, especially for myocardial perfusion [4–10] and brain [11–13] diagnosis. In simultaneous multi-radionuclide imaging, primary gamma rays emitted from each radionuclide are distinguished by their energy, ideally. However, some gamma ray photons lose energy due to matter interaction caused, for example, by the photoelectric effect or Compton scattering. Moreover, some gamma ray photons are detected as different-energy photons due to the system’s limited energy resolution. These phenomena can cause crosstalk between the radionuclide energy spectra. To enable accurate simultaneous multi-radionuclide imaging, a detector with good energy resolution or a complicated scatter and crosstalk correction method is necessary. Therefore, simultaneous multi-radionuclide imaging is not generally conducted in clinical settings even though it is attractive. The recent development of several cardiac-SPECT systems using solid-state detectors [7, 14, 15] with very good energy resolution has led to simultaneous multi-radionuclide imaging [4–6] recapturing the spotlight.

Our group previously developed a prototype brain imaging SPECT system with two heads that uses cadmium telluride (CdTe) solid-state detectors [16] and two wide-aperture parallel-hole collimators, which we call “4-pixel matched collimators (4-PMCs)” [17]. Pixelated CdTe detectors with a pixel pitch of 1.4 mm have very good intrinsic spatial resolution (1.7 mm) and very good energy resolution (6.6 % at 141 keV) [18]. The system performance was evaluated for brain perfusion single-radionuclide imaging [16]. The results showed that the prototype CdTe detector-based SPECT system can provide good spatial resolution and high-contrast images with good scatter rejection.

In simultaneous dual-radionuclide imaging, even if the energy resolution of the CdTe detectors is very good, down-scatter is unavoidable. Furthermore, scatter within the crystals, k-escape, and incomplete charge collection can cause a considerable fraction of the primary gamma rays to be measured as lower energy events [4–8]. An iterative deconvolution-based scatter and crosstalk correction method for simultaneous dual-radionuclide imaging and a solid-state detector SPECT system have been developed [4]. The method improves the contrast in simultaneous Tc-99m and Tl-201 imaging using CdZnTe-based SPECT. The feasibility of using this method for simultaneous Tc-99m and I-123 cardiac imaging has been investigated [8].

Other groups have also developed sophisticated scatter correction methods for simultaneous Tc-99m and I-123 cardiac imaging [5, 6]. They modelled the physical phenomena of scatter in detail and incorporated the model into a maximum likelihood expectation maximization (MLEM)-based correction method. Their approach achieved good correction accuracy. However, the required MLEM algorithm represents a significant computational burden, and the relatively many parameters are needed to be optimized.

In this study, we developed a fast and simple energy-based scatter correction method using five energy windows for simultaneous Tc-99m and I-123 brain imaging. This five energy window (FiveEW) method uses a modified triple-energy window (TEW) method based on the TEW method reported by Ichihara et al. [19]. This TEW method is widely used and has a long history with a certain amount of clinical usage. In addition, energy-window-based scatter correction methods like TEW ones can compensate for scatter from out of field of view (FOV), which is difficult for simulation-based correction. However, overcorrection by TEW method in simultaneous dual-radionuclide imaging has been reported [6]. Our aims were to evaluate the quantitative accuracy of simultaneous Tc-99m and I-123 brain studies with a CdTe-SPECT system using the FiveEW method and to make simultaneous dual-radionuclide imaging more practical and easier to conduct. The physical performance of simultaneous dual-radionuclide imaging was evaluated experimentally by using two phantom studies. We also demonstrated the first clinical application of Tc-99m human serum albumin diethylene triamine pentaacetic acid (HSA-D) and I-123 *N*-isopropyl-4-iodoamphetamine hydrochloride (IMP) in a dual-radionuclide study of five healthy volunteers.

## Methods

### Prototype solid-state brain imaging SPECT system

Our prototype system uses CdTe solid-state detectors and two 4-PMCs for high-sensitivity brain imaging [17]. The system has two detector heads, each consisting of 192 × 96 detector pixels with a pitch of 1.4 mm, so the FOV of each detector head is 268 mm × 134 mm (tangential × axial). The size of each detector pixel is 1.2 mm × 1.4 mm × 5 mm. The collimator hole size of each 4-PMC is matched to a 2 × 2 array of detector pixels. That is, four detectors are placed in one hole of 4-PMC. The hole size, pitch, and length of 4-PMC are 2.4, 2.8, and 26 mm, respectively. The rotation radius of each detector head is 130 mm. SPECT acquisition is performed in continuous rotation mode in which each detector head is rotated 360° over 3 min. The intrinsic energy resolution (FWHM) of the CdTe detectors is 6.6 % at 141 keV. The long-term stability of the energy resolution of the detectors is ensured by periodically resetting the bias [20] every 3 min as the detector heads rotate. No significant variances in the spectral peak position and energy resolution of the detectors were observed for over a year [18]. This very good energy resolution enables the photopeaks of Tc-99m (141 keV) and I-123 (159 keV) to be distinguished. In simultaneous Tc-99m and I-123 dual-radionuclide imaging, five kinds of energy windows are used to correct for scatter and crosstalk, as described below.

### Scatter and crosstalk correction for simultaneous dual-radionuclide imaging

**Fig. 1:**
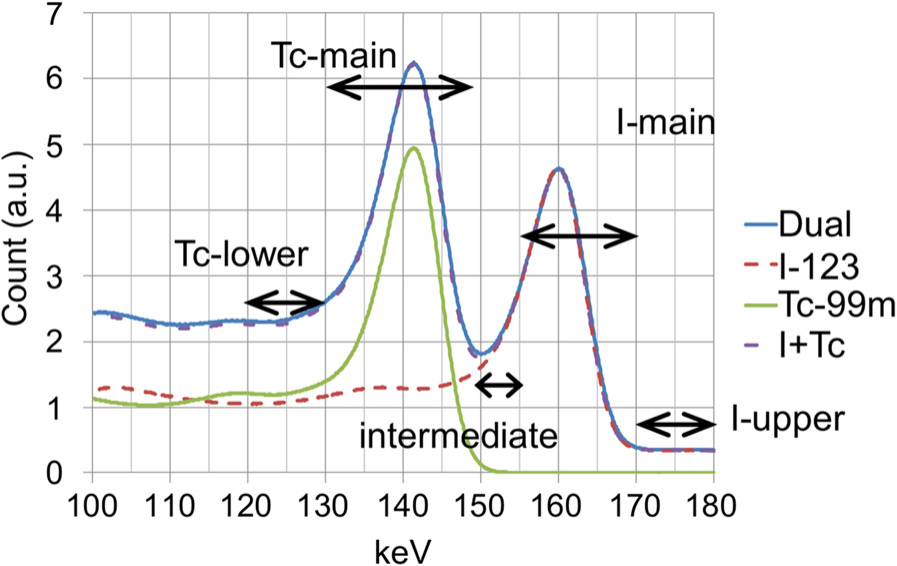
Measured energy spectra for Tc-99m and I-123 and for simultaneous dual-radionuclide scan: measured energy spectra for Tc-99m, I-123, simultaneous Tc-99m and I-123 dual-radionuclide scanning (dual), and sum of Tc-99m and I-123 spectra measured separately (I + Tc). Energy resolution of CdTe solid-state detector was good enough to distinguish photopeaks of Tc-99m (141 keV) and I-123. Settings for five energy windows are labelled. No differences in shapes between “dual” and “I + Tc” were observed

The FiveEW scatter and crosstalk correction method we developed for simultaneous Tc-99m and I-123 imaging uses five energy windows: Tc-lower, Tc-main, shared sub-window of Tc-upper and I-lower, I-main, and I-upper (Table 1 and Fig. 1). The position and width of the energy windows were chosen so that the lower sub-window of I-123 does not contain Tc-99m counts, each main window contains the photopeak energy range, and the widths of other sub-windows are 10 keV to reduce statistical error. Another main window for a higher photopeak of I-123 (529 keV) was not used in this study; instead, the down-scatter from the photopeak is dealt with as scatter in the upper energy window for I-123. The scatter in each radionuclide main window is corrected using a TEW-based scatter correction method using a main window and two sub-windows related to the radionuclide of interest.

**Table 1:** Energy window settings

Window	keV
Tc-lower	120–130
Tc-main	130–148
Intermediate (Tc-upper and I-lower)	150–155
I-main	155–170
I-upper	170–180

The TEW method was modified to compensate for excess scatter correction. It reduces scatter simply with a low computational burden. The algorithm for the TEW method is

1 $$ \mathrm{T}\mathrm{E}\mathrm{W}\left({E}_L,{E}_M,{E}_U\right)={E}_M-\left(\alpha \cdot {E}_L+\beta \cdot {E}_U\right) $$

Where

2 $$ \alpha =\frac{C_U-{C}_M}{C_U-{C}_L}\times \frac{W_M}{W_L} $$

3 $$ \beta =\frac{C_M-{C}_L}{C_U-{C}_L}\times \frac{W_M}{W_U} $$

The *E*_*x*_ represents the matrix for the count detected in energy window *x* (i.e. projection data for energy window *x*). The *C*_*x*_ represents the centre energy of energy window *x*. TEW(*E*_*L*_,*E*_*M*_,*E*_*U*_) represents the scatter-corrected count matrix for energy window *M* calculated from *E*_*L*_, *E*_*M*_, and *E*_*U*_ using the TEW method. The *W*_*x*_ represents the width of energy window *x. L* represents the lower window, *M* represents the main window, and *U* represents the upper window. It is assumed that *E*_*L*_ and *E*_*U*_ for this TEW method do not contain any primary gamma ray counts. With a pixelated solid-state detector, however, a certain number of primary gamma rays are detected as lower energy gamma rays due to incomplete charge collection and inter-pixel scatter [4–6]. Therefore, *E*_*L*_ contains some primary counts (photopeak spillover), and the TEW method subtracts the primary counts from the main window, E_M_.

**Fig. 2:**
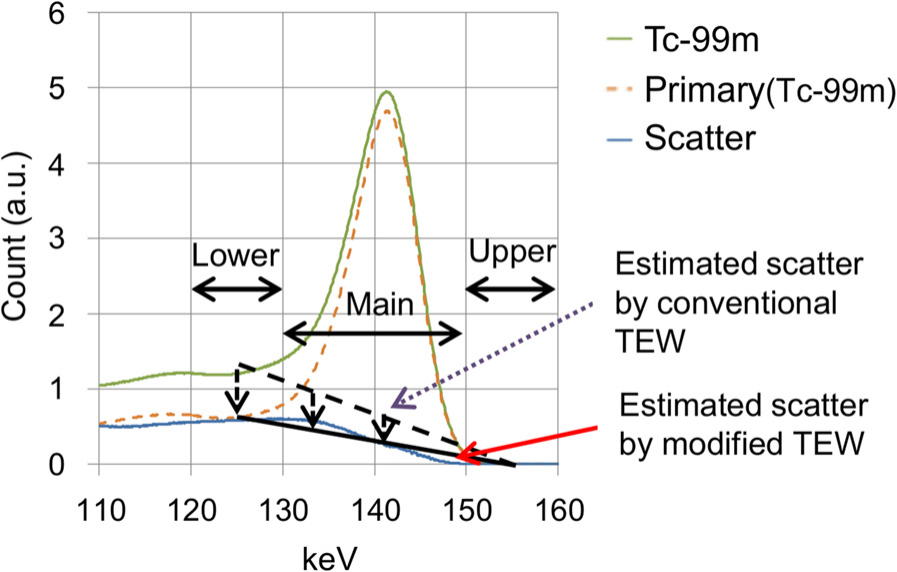
Concept of modified TEW method: spectra for Tc-99m and primary (Tc-99m) were obtained from phantom measurement and scatter-free radiation source measurement. Spectrum for “scatter” was calculated by subtracting “primary (Tc-99m)” from “Tc-99m.” Conventional TEW method and modified TEW method are described in Eqs. 1 and 6, respectively. Amount of estimated scatter was reduced to acceptable level by taking into account fraction of primary count in lower sub-window

Our modified TEW method uses the detector response (DR) for primary gamma rays (Fig. 2). The response is the energy spectrum of the primary gamma rays obtained from pre-measurement of a scatter-free radiation source. The detector response of our CdTe detectors is stable enough to be used to calculate the sensitivity ratio of each energy window as a constant ratio [18]. By using these sensitivity ratios, we can estimate the primary counts for the lower window:

4 $$ {P}_L=r\cdot {P}_M=r\cdot \mathrm{T}\mathrm{E}\mathrm{W}\left({E}_L-{P}_L,{E}_M,{E}_U\right) $$

where *r* is the sensitivity ratio calculated by dividing the detector response for the primary gamma rays of the lower window by that of the main window. The *P*_*L*_ and *P*_*M*_ are the primary counts for the lower and main windows, respectively. In this equation, the TEW method is assumed to correct for scatter properly if the input counts of the sub-windows do not contain primary counts. Because Eq. 1 is a simple and linear function, Eq. 4 is solved in the form

5 $$ \mathrm{T}\mathrm{E}\mathrm{W}\left({E}_L-{P}_L,{E}_M,{E}_U\right)={E}_M-\left(\alpha \cdot \frac{E_L-r\cdot {E}_M+\beta \cdot r\cdot {E}_U}{1-\alpha \cdot r}+\beta \cdot {E}_U\right) $$

On the right side of Eq. 5, the first term is the measured count matrix and the second term is the estimated scattered count matrix. Equation 5 estimates the counts corresponding to primary gamma rays detected in the sub-windows, subtracts them from the total counts for the sub-windows, and then finally corrects the scatter for the main window (Fig. 2). Equation 5 can be represented as

6 $$ \mathrm{T}\mathrm{E}\mathrm{W}\left({E}_L-{P}_L,{E}_M,{E}_U\right)=\frac{E_M-\alpha {E}_L-\beta {E}_U}{1-\alpha r}=\frac{\mathrm{TEW}\left({E}_L,{E}_M,{E}_U\right)}{1-\alpha r} $$

To reduce statistical fluctuation of the estimated scatter, a filter *f* is applied to the scatter term in Eq. 5. The filter was a Butterworth filter with order 8 and a cut-off frequency 0.056 cycles/pixel. The proposed scatter correction method, TEW method with detector response (TEWDR), is then given by

7 $$ \mathrm{TEWDR}\left({E}_L,{E}_M,{E}_U\right)={E}_M-\left(\alpha \cdot \frac{E_L-r\cdot {E}_M+\beta \cdot r\cdot {E}_U}{1-\alpha \cdot r}+\beta \cdot {E}_U\right)\otimes f, $$

where TEWDR(*E*_*L*_,*E*_*M*_,*E*_*U*_) represents the scatter-corrected count matrix of energy window *M* calculated from *E*_*L*_, *E*_*M*_, *and E*_*U*_.

For quantitative simultaneous dual-radionuclide imaging, the FiveEW method uses TEWDR to correct for scatter in each radionuclide image. The count for the scattered events in I-main is corrected for by using the counts for the I-lower, I-main, and I-upper windows.

The contamination from I-123 to the Tc-related windows is calculated using

8 $$ {\mathrm{CI}}_x={S}_x/{S}_{\mathrm{I}\hbox{-} \mathrm{main}}\cdot {E}_{\mathrm{I}\hbox{-} \mathrm{main}} $$

where CI_*x*_ represents the contamination count from I-123 to Tc-related energy window *x*. The *S*_*X*_ and *S*_I-main_ represent the sensitivities of energy window *x* and I-main for I-123, respectively. The *S*_*X*_ is the normalized total count of the detector response for primary events from I-123 in window *x*. The *E*_I-main_, the count matrix for the I-main window, contains primary and scattered counts from I-123. Therefore, CI_*x*_ contains the contamination count from I-123 due to limited energy resolution, incomplete charge collection, inter-pixel scatter, and down-scatter from higher photopeaks such as 529 keV but does not contain the down-scatter from I-main. There are two reasons for using I-main for crosstalk correction instead of the primary counts of I-123 (i.e. scatter-corrected I-main). One is that crosstalk occurs due to not only down-scatter but also limited energy resolution and incomplete charge collection. Some scattered photons from photopeaks higher than 159 keV enter the detector with an energy within that of the I-main window. The other reason is that the distributions of I-123 and Tc-99m are different. If the primary counts distribution of I-123 was subtracted for crosstalk correction, the considerable amount of scatter caused by photons coming from I-123 would remain in the energy windows for Tc-99m. It is difficult for the TEW and TEWDR methods to distinguish the I-123 and Tc-99m counts. Therefore, to reduce the amount of scatter from I-123, I-main was used for crosstalk correction. The calculated contamination is subtracted from the Tc-related windows. Finally, the count of scattered events including the residual scatter from I-123 in Tc-main is corrected for using the counts for the Tc-lower, Tc-main, and Tc-upper windows. In short, FiveEW correction for scatter and crosstalk can be represented as Eq. 9. The intermediate window (*E*_intermediate_) acts as *E*_*U*_ and *E*_*L*_ for Tc-99m and I-123, respectively. Eq. 9 is applied pixel by pixel in the projection data in the scatter correction procedure.

9 $$ \begin{array}{l}\mathrm{FiveEW}\left({E}_{\mathrm{Tc}\hbox{-} \mathrm{lower}},{E}_{\mathrm{Tc}\hbox{-} \mathrm{main}},{E}_{\mathrm{intermediate}},{E}_{\mathrm{I}\hbox{-} \mathrm{main}},{E}_{\mathrm{I}\hbox{-} \mathrm{upper}}\right)\\ {}\kern3.96em =\left\{\begin{array}{c}\hfill \mathrm{TEWDR}\left({E}_{\mathrm{intermediate}},{E}_{\mathrm{I}-\mathrm{main}},{E}_{\mathrm{I}-\mathrm{upper}}\right),\kern0.5em \mathrm{f}\mathrm{o}\mathrm{r}\ \mathrm{I}\hbox{-} 123\hfill \\ {}\hfill \mathrm{TEWDR}\left({E}_{\mathrm{Tc}\hbox{-} \mathrm{lower}}-\mathrm{C}{\mathrm{I}}_{\mathrm{Tc}\hbox{-} \mathrm{lower}},{E}_{\mathrm{Tc}-\mathrm{main}}-\mathrm{C}{\mathrm{I}}_{\mathrm{Tc}\hbox{-} \mathrm{main}},{E}_{\mathrm{intermediate}}-\mathrm{C}{\mathrm{I}}_{\mathrm{Tc}\hbox{-} \mathrm{upper}}\right)\hfill \\ {}\hfill, \kern0.5em \mathrm{f}\mathrm{o}\mathrm{r}\ \mathrm{T}\mathrm{c}\hbox{-} 99\mathrm{m}\hfill \end{array}\right.\end{array} $$

### Image reconstruction and attenuation correction

Following scatter correction, the projection data are reconstructed by ordered subset expectation maximization (OSEM) [21] including point spread function (PSF) and attenuation correction. The PSF is obtained by ray-tracing simulation [16]. The attenuation factor of the gamma rays passing through an object is approximated by an exponential function of the line integral from an image voxel to a detector pixel in an attenuation map. The contour of the attenuation map is generated by tracing the edge of an object in the projection image and then assigning the region within the contour a uniform attenuation coefficient (0.015 mm^−1^ for Tc-99m; 0.0146 mm^−1^ for I-123) [22]. Rotation-based forward and backward projections are performed using the PSF and attenuation factor [23].

The projection image matrix size (tangential × axial) is 256 × 96 (pixel size = 1.4 mm × 1.4 mm). The number of projection images is 120 over 360°. The reconstructed image matrix size (*x* × *y* × *z*) was 256 × 256 × 96 (voxel size = 1.4 mm × 1.4 mm × 1.4 mm) when the numbers of subsets and iterations were 30 and 20, respectively. The reconstructed image was smoothed using a 14-mm FWHM Gaussian filter.

### Preliminary evaluation of scatter correction accuracy using Monte Carlo simulation

**Fig. 3:**
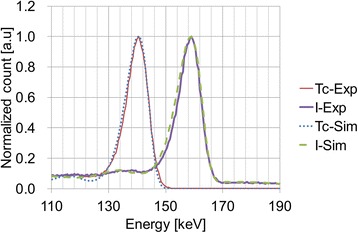
Simulated and measured energy spectra for Tc-99m and I-123: choosing parameters that fit these spectra resulted in energy resolution and tailing effect being well modelled

Two kinds of Monte Carlo simulation using Geant4 [24, 25] with a line source and a cylindrical phantom were performed to validate the proposed scatter correction. The simulation generated detected energy histogram for each detector without concerning energy resolution. The detected energy histograms for primary and scattered photons were generated separately. The tailing effect of the solid-state detector and energy resolution due to incomplete charge collection and statistical fluctuation, respectively, were simulated from the detected energy histogram using the Hecht equation [26–28], Fano factor [26, 27, 29], and a constant representing electrical noise [26, 27]. The photon interaction positions in the detector used for calculating the Hecht equation were randomly assigned in accordance with a Gaussian distribution. Two kinds of planar images of Tc-99m and I-123 line sources with a diameter of 10 mm and a length of 100 mm were simulated for spectral fitting between the simulation and experimental results (Fig. 3). Two kinds of planar images of a cylindrical phantom with a diameter of 200 mm and a length of 200 mm were simulated to validate the proposed scatter correction method. The radionuclides filled in the cylindrical phantom were Tc-99m and I-123. Simultaneous Tc-99m and I-123 planar imaging data were generated by summing the detected energy histograms of Tc-99m and I-123 for the cylindrical phantom while varying the Tc-99m:I-123 photopeak count ratio for various Tc-99m:I-123 photopeak count ratios: 1:9, 2:8, 3:7, 4:6, 5:5, 6:4, 7:3, 8:2, and 9:1. All planar images were corrected for scatter by using the proposed FiveEW method and FiveEW method using TEW instead of TEWDR (FiveEW-woDR). The percentage errors between the scatter-corrected count and the primary count for the planar images were evaluated.

### Evaluation of scatter correction accuracy using phantoms

As mentioned, we conducted two phantom experiments with our prototype CdTe-SPECT system to evaluate the quantitative accuracy of simultaneous dual-radionuclide imaging.

**Fig. 4:**
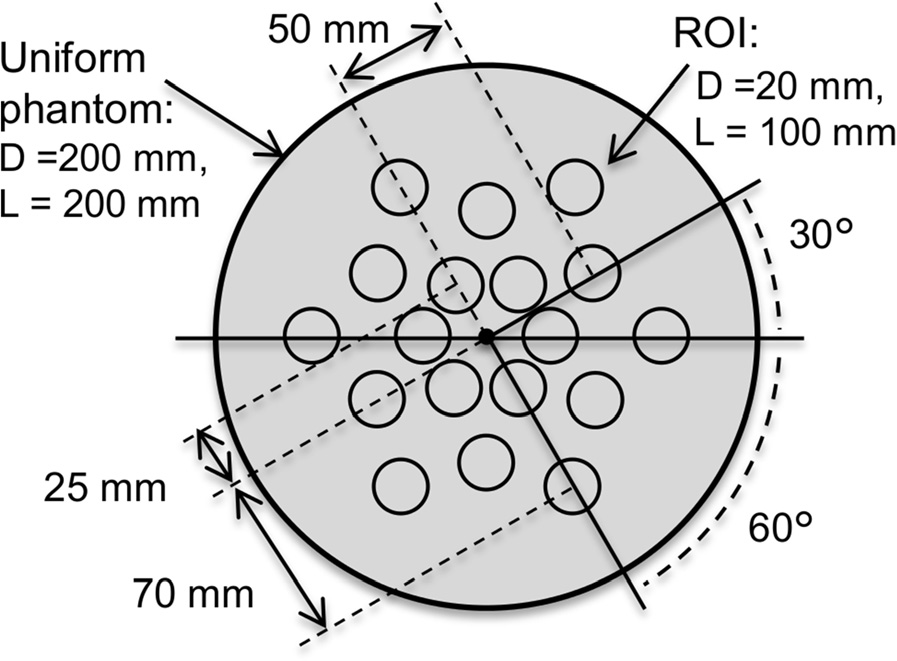
Uniform phantom and ROI settings: distances from centre of phantom to ROI centres were 25, 50, and 70 mm. Each ROI had same radius of rotation; they were placed every 60°

A uniform phantom experiment was conducted to evaluate the quantitative accuracy of each radionuclide activity measurement. The phantom was a circular polyethylene cylinder with an inside diameter of 200 mm, an outside diameter of 203 mm, and a length of 200 mm. The phantom was filled with Tc-99m (112 MBq) and scanned for 21 min (single acquisition). Then, I-123 (77 MBq) was additionally injected into the phantom, and the phantom was scanned again for 21 min (dual acquisition). The scanned projection data for the single acquisition were corrected for scatter by using a TEW and the developed TEWDR methods. The scanned projection data for the dual acquisition were corrected for scatter by using the FiveEW and FiveEW-woDR methods. Then, after decay correction, the Tc-99m image for the single acquisition and the Tc-99m and I-123 images for the dual acquisition were reconstructed from the scatter-corrected projection data by OSEM as mentioned above. The mean voxel values for 18 regions of interest (ROIs) placed inside the reconstructed phantom images were compared. The ROIs were cylinders with a diameter of 20 mm and a length of 100 mm and were placed as shown in Fig. 4. Cross-calibration factors for each correction method (including no correction) and each radionuclide were calculated by dividing the true activities measured using a well counter by the mean voxel values with single acquisition for use in the following phantom experiment.

**Fig. 5:**
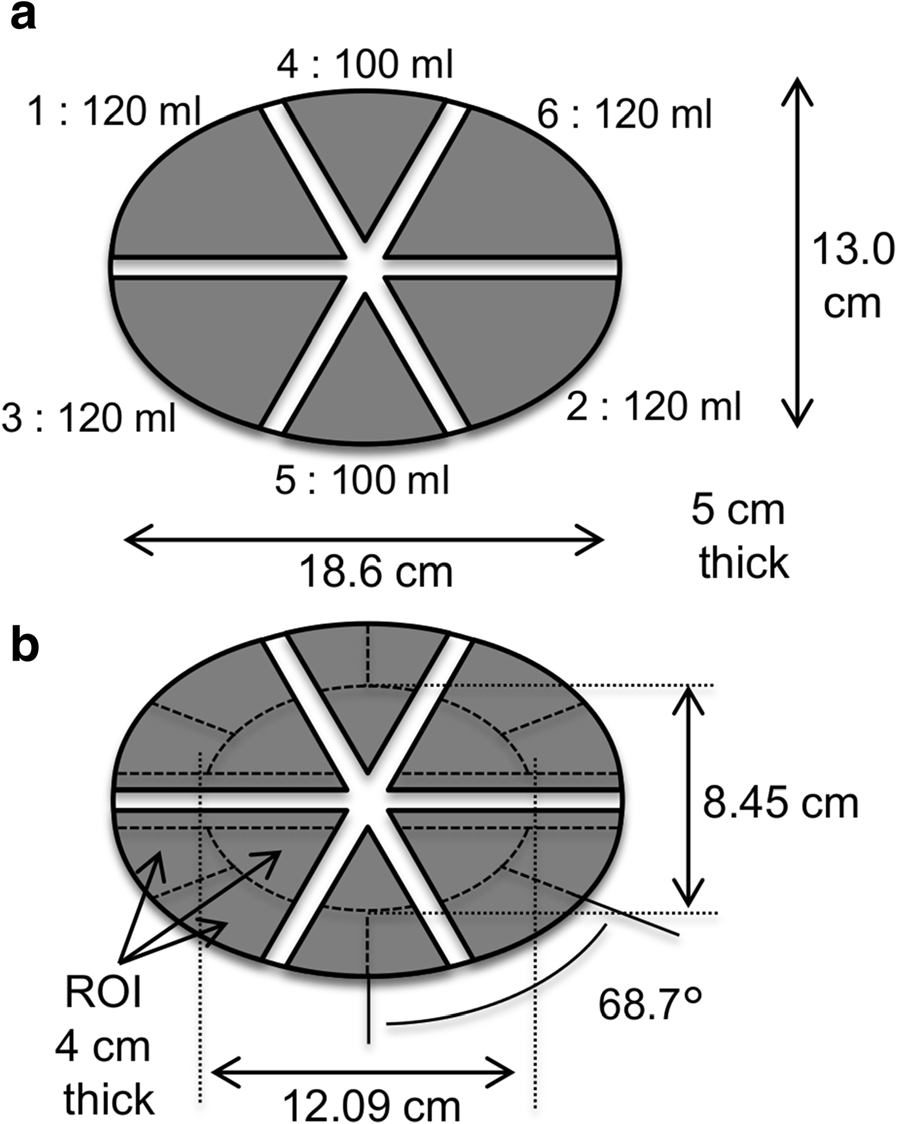
Six-compartment phantom (**a**) and ROI setting (**b**): three kinds of ROI were set in each compartment

A six-compartment phantom experiment using an IB-10 brain phantom (Kyoto Kagaku Co., Ltd.) was conducted to evaluate the quantitative accuracy of the linearity of each radionuclide activity. The six isolated compartments in the IB-10 brain phantom (Fig. 5a) were filled with different activities (Table 2). This phantom is a polyethylene elliptic cylinder with an outside major diameter of 18.6 cm, a minor diameter of 13 cm, and a length of 5 cm. The phantom was scanned for 60 min (dual acquisition). The measured list data were divided into five frames (12 min/frame) and then converted to five kinds of projection data. The projection data were corrected for scatter by using the FiveEW and FiveEW-woDR methods. The Tc-99m and I-123 images were then reconstructed from the scatter-corrected projection data by OSEM. The mean voxel values for the 18 ROIs shown in Fig. 5b of the reconstructed phantom images for each frame were measured. The mean ROI activities estimated using the cross-calibration factors measured in the uniform phantom experiment were compared with the actual radiation activity to evaluate count measurement reproducibility between single and dual acquisitions.

**Table 2:** Activities of six-compartment phantom

Compartment	Volume (ml)	Tc-99m (kBq/ml)	I-123 (kBq/ml)	Target ratio of Tc:I
1	120	295	95	3:1
2	120	0	372	0:4
3	120	411	0	4:0
4	100	201	186	2:2
5	100	0	0	0:0
6	120	96	278	1:3

### Evaluation of scatter correction accuracy using healthy volunteers

To evaluate the feasibility of a dual-radionuclide clinical study, we conducted a study of healthy volunteers in which we acquired cerebral blood volume (CBV) and cerebral blood flow (CBF) images simultaneously using Tc-99m HSA-D and I-123 IMP (Nihon Medi-Physics Co., Ltd) and the prototype CdTe-SPECT system.

Five healthy male volunteers participated. The institutional review boards of both the Hokkaido University Graduate School of Medicine and the Central Research Laboratory of Hitachi Ltd. approved this study and all volunteers gave written informed consent. Their ages ranged from 20 to 49, their body mass indices ranged from 22.8 to 32.0, and their mean body mass index was 25.8.

CBV images were acquired 5 min after Tc-99m HSA-D injection (avg. 327 MBq) for 21 min (single acquisition). Following image acquisition, I-123 IMP was injected (avg. 167 MBq) intravenously, and CBF and CBV images were acquired simultaneously 20 min after injection (dual acquisition) for 21 min. The scanned projection data were corrected for scatter by using the FiveEW method. Then, the single-acquisition CBV images and the dual-acquisition CBV and CBF images were reconstructed from the scatter-corrected projection data by OSEM after decay correction. The whole brain counts of the CBV images for single and dual acquisitions were compared, and the intra-class correlation coefficients between the single- and dual-acquisition images were calculated. Two experienced nuclear physicians visually inspected the images and evaluated their quality using a five-point scale (1 = poor to 5 = excellent). They were unaware of the type of image being viewed, and the images were presented in random order.

## Results

### Preliminary evaluation using simulation

Table 3 shows the absolute percentage errors between the scatter corrected and the primary counts for the simulated planar images. The FiveEW method used the energy spectra of the primary images for Tc-99m and I-123 as the DR. “FiveEW-woDR” represents the FiveEW method using the TEW method instead of TEWDR. For single acquisition, the percentage errors were improved by the scatter correction with TEWDR. The percentage errors were due to scatter for none (no correction) and overcorrection for FiveEW-woDR and FiveEW in Table 3. For dual acquisition, the percentage error for I-123 was lower with FiveEW than with FiveEW-woDR but was still worse than that with no correction. This is because the lower sub-window for I-123 was placed within the photopeak energy range. On the other hand, the percentage errors for Tc-99m were improved for FiveEW and FiveEW-woDR; that for FiveEW-woDR was slightly lower than that for FiveEW.

**Table 3:** Percentage errors between scatter corrected count and primary count

RI of interest	Tc-99m			I-123		
Acquisition (photopeak count ratio)	None	FiveEW-woDR	FiveEW	None	FiveEW-woDR	FiveEW
Single (%)	25.6	4.1	2.4	14.3	72.6	39.9
Dual (Tc:I = 1:9) (%)	700.4	27.0	35.5	14.3	72.6	39.9
Dual (Tc:I = 2:8) (%)	325.5	9.7	17.1	14.3	72.6	39.9
Dual (Tc:I = 3:7) (%)	200.5	4.0	11.0	14.3	72.6	40.0
Dual (Tc:I = 4:6) (%)	138.1	1.1	7.9	14.3	72.6	40.0
Dual (Tc:I = 5:5) (%)	100.6	0.6	6.1	14.3	72.6	40.0
Dual (Tc:I = 6:4) (%)	75.6	1.8	4.8	14.3	72.7	40.1
Dual (Tc:I = 7:3) (%)	57.7	2.6	4.0	14.3	72.7	40.2
Dual (Tc:I = 8:2) (%)	44.3	3.2	3.3	14.3	72.8	40.5
Dual (Tc:I = 9:1) (%)	33.9	3.7	2.8	14.3	73.1	41.2
Dual (mean ± SD) (%)	186.3 ± 213.8	6.0 ± 8.3	10.3 ± 10.5	14.3 ± 0	72.7 ± 0.2	40.2 ± 0.4

Values are percentage errors between scatter corrected count and primary count

*SD* standard deviation

### Evaluation using phantoms

The results of the uniform phantom study are summarized in Table 4, where the means are the mean voxel values for all ROIs, and the standard deviations are the standard deviations of the voxel values for all ROIs. For single acquisition of Tc-99m images, using DR (i.e. TEWDR) gave a higher mean ROI value than without DR (i.e. TEW). For dual acquisition of Tc-99m and I-123 images, the FiveEW using DR method also gave a higher mean ROI value than without using DR (i.e. FiveEW-woDR method). The mean voxel values corrected using DR were more than 1.1 times those corrected without using DR. This means that using DR for scatter correction improved the overestimation of scatter compared to using TEW without DR. The percentage differences between single and dual acquisitions were 5.7 and 4.3 %, corresponding to scatter correction and crosstalk correction using DR and without using DR, respectively.

**Table 4:** Results of uniform phantom study

Radionuclide	Acquisition	Evaluation indicator	No correction	Corrected without DR	Corrected using DR	DR/no DR
Tc-99m	Single	ROI value	260.5 ± 12.9	183.7 ± 17.8	204.2 ± 19.4	1.11
	Dual	ROI value	342.3 ± 16.9	188.2 ± 16.6	214.9 ± 18.6	1.14
	Dual vs. single	PD (%)	27.1 ± 3.2	4.3 ± 3.2	5.7 ± 4.0	1.32
I-123	Dual	ROI value	111.2 ± 8.1	58.2 ± 8.0	75.3 ± 9.4	1.29

Values are mean ± standard deviation

*PD* percentage difference for each ROI value between single and dual acquisitions

**Fig. 6:**
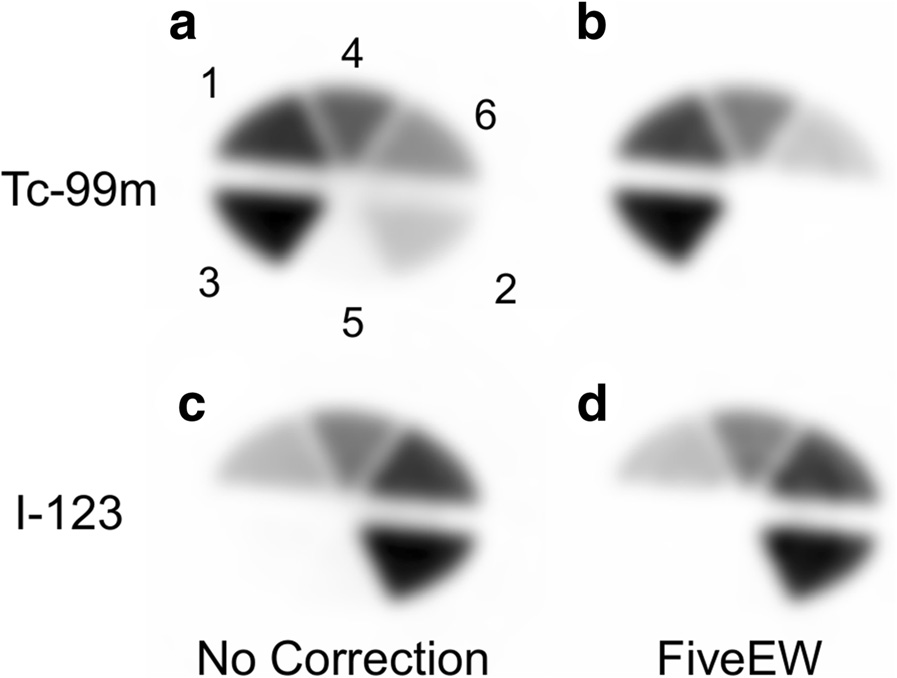
Six-compartment phantom images: simultaneous dual-radionuclide images of mixture of Tc-99m and I-123. Tc-99m images with no correction (**a**) and corrected by FiveEW (**b**); I-123 images with no correction (**c**) and corrected by FiveEW (**d**). After scatter correction by FiveEW method, contamination from I-123 in compartment 2 clearly disappeared

**Fig. 7:**
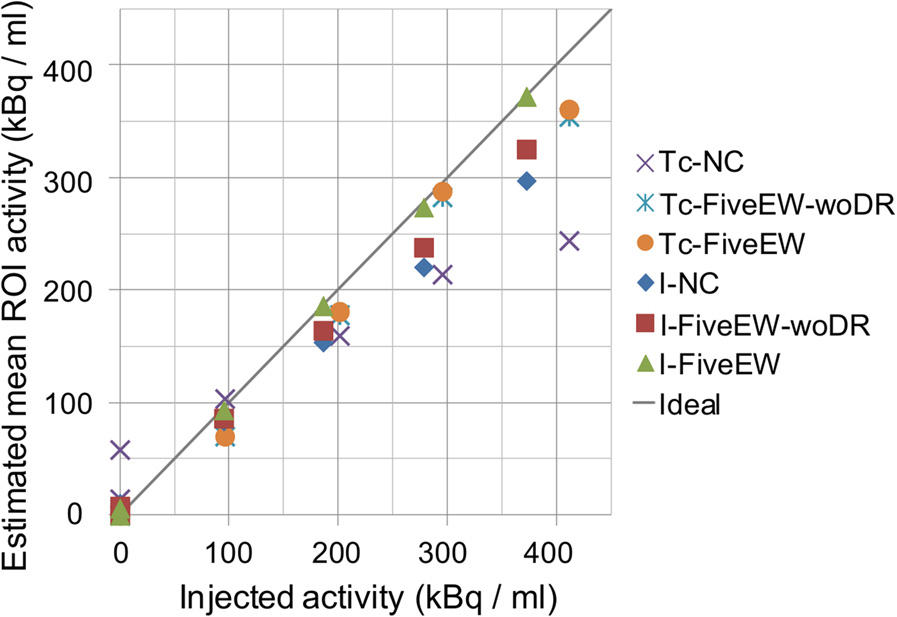
Linearity of activities of Tc-99m and I-123 six-compartment phantom: scattering diagram of estimated mean ROI activities for six compartments in image with no correction (NC) and corrected by FiveEW and FiveEW-woDR methods. Tc and I indicate main energy window for radionuclide of interest

The results of the six-compartment phantom study are shown in Figs. 6 and 7 and Table 5. Figure 6 shows the reconstructed images without scatter correction (NC) and with scatter correction using the FiveEW method. Figure 7 shows the linearity of activity measurement. The horizontal axis is an injected activity, and the vertical axis is an estimated mean activity of the corresponding compartment. Table 5 shows the relative percentage difference between the standardized hot ROI values and the injected activity ratios, the percentage difference (PD) between the hot ROI values and the injected activity, and the residual scatter (RS) of the cold ROI values. The standardized hot ROI values were calculated by dividing the hot ROI values by the ROI value for a compartment in which the activity of the radionuclide of interest was the highest (i.e. compartment 4 or 2 for Tc-99m or I-123, respectively). The injected activity ratio was the radiation dose ratio of each hot ROI to the ROI for a compartment in which the activity of the radionuclide of interest was the highest (i.e. compartment 4 or 2 for Tc-99m or I-123, respectively). The cold and hot ROIs refer to the ROIs for the compartments for which the injected activity was zero and not zero, respectively, for each radionuclide. RS was defined as the mean cold ROI value divided by the mean hot ROI value.

**Table 5:** Results for six-compartment phantom images

Radionuclide	Tc-99m			I-123		
Correction	None	FiveEW-woDR	FiveEW	None	FiveEW-woDR	FiveEW
Relative PD (%)	27.3 ± 20.4	7.9 ± 6.6	8.4 ± 7.3	4.5 ± 3.7	2.7 ± 2.0	2.4 ± 1.8
PD (%)	28.3 ± 16.1	15.6 ± 10.2	14.4 ± 11.0	19.3 ± 4.9	12.8 ± 3.3	2.3 ± 1.8
RS (%)	20.1 ± 12.4	1.3 ± 1.4	1.3 ± 1.4	3.4 ± 2.5	2.1 ± 2.1	1.3 ± 1.6

Values are mean ± standard deviation

*PD* percentage difference for each ROI value between hot ROI values and injected activity, *RS* residual scatter defined as mean cold ROI value divided by mean hot ROI value

No contamination from Tc-99m was observed in any of the I-123 images. The percentage difference with no correction was worse than those with scatter correction, because the shapes of the uniform phantom for measuring the cross-calibration factors and the six-compartment phantom were different (meaning that the scatter fractions and scatter distributions in these tests were different). The scatter and crosstalk for the Tc-99m images were well corrected by the FiveEW and FiveEW-woDR methods (Fig. 6 and Table 5). The relative PDs for the two methods were similar (Table 5). Both methods improved the qualitative accuracy of simultaneous dual-radionuclide imaging. For I-123, the FiveEW method improved the mean PD and mean RS compared to the FiveEW-woDR method. For Tc-99m, the mean PDs and mean RSs of the two methods were similar. The FiveEW method improved the quantitative accuracy of simultaneous dual-radionuclide imaging, especially for the I-123 images.

### Evaluation using healthy volunteers

**Fig. 8:**
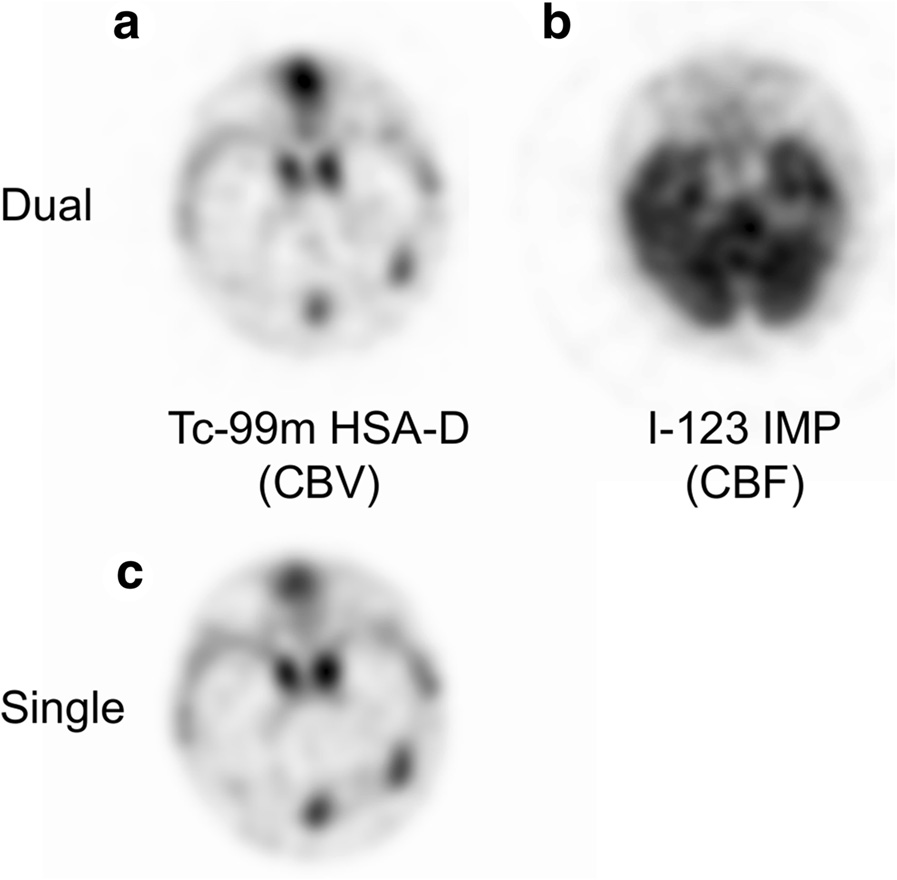
Results of a study of healthy volunteers: images for simultaneous Tc-99m HAS-D (**a**) and I-123 IMP (**b**) dual acquisition and Tc-99m HAS-D (**c**) single acquisition for volunteer 3. Scatter and crosstalk were corrected by FiveEW method

The results of the study of healthy volunteer are shown in Table 6 and Fig. 8. The mean PD for the Tc-99m HSA-D CBV images was 4.5 ± 3.4 % (Table 6). The mean intra-class correlation coefficient between the single- and dual-acquisition CBV images for each volunteer was 0.99. The qualities of the CBV single- and dual-acquisition images were consistent for all volunteers. Figure 8 shows a single-acquisition CBV image and dual-acquisition CBV and CBF images for volunteer 3. The means of the visual scores for the Tc-99m single- and dual-acquisition CBV images and the I-123 dual-acquisition CBF image were 5, 5, and 4.6 (±0.54, S.D.), respectively (1 = poor to 5 = excellent).

**Table 6:** Comparison of single and dual acquisitions for CBV images

Volunteer	Percentage difference (%)
1	4.5
2	9.9
3	3.4
4	0.5
5	4.4
Mean ± SD	4.5 ± 3.4

*SD* standard deviation

## Discussion

The simultaneous Tc-99m and I-123 dual-radionuclide imaging method we have developed uses a pixelated solid-state detector SPECT system and an energy-window-based simple scatter and crosstalk correction method. The energy resolution of the CdTe solid-state detector is good enough to distinguish the photopeaks of Tc-99m (141 keV) and I-123 (159 keV) (Fig. 1). This enables the scanner to provide an I-123 image without Tc-99m contamination in simultaneous dual-radionuclide imaging. Therefore, the I-123 images provided by the CdTe-SPECT are reliable even without crosstalk correction for both simultaneous and sequential Tc-99m and I-123 scanning on the same day. This is advantageous for using a simple scatter and crosstalk correction method like our five energy window method (FiveEW method). The CdTe solid-state detector also provides a stable energy spectrum. This is very important as it enables the use of the detector response for the primary gamma rays for scatter and crosstalk correction.

FiveEW is a modified TEW (TEWDR)-based scatter and crosstalk correction method designed for simultaneous Tc-99m and I-123 imaging. TEWDR uses the detector response for the primary gamma rays and estimates the counts corresponding to the primary gamma rays in sub-windows to avoid overcorrection. It increased the counts for the scatter-corrected main window over 10 % compared to TEW (Table 4). The effect of using detector responses for the primary gamma rays in the FiveEW method was better for the I-123 images than the Tc-99m ones (Tables 3 and 5). This is because the mean detector responses for the primary gamma rays or primary counts in the sub-windows for I-123 were greater than those for Tc-99m because the sub-windows for I-123 were narrower and nearer to the photopeak than those for Tc-99m (Fig. 1).

Comparison of the results of the simulation and experiments shows that the percentage errors between scatter corrected I-123 images and primary I-123 images were not so good (72.7 and 40.2 % for FiveEW-woDR and FiveEW, respectively, Table 3), but the percentage differences between the ROI value for I-123 images and the injected activity in the six-compartment phantom experiment were good (12.8 and 2.3 % for FiveEW-woDR and FiveEW, respectively, Table 5). The causes of the percentage errors in simulation were scatter for none (no correction) and overcorrection for FiveEW-woDR and FiveEW in Table 3. The differences in the six-compartment phantom experiment (Table 5) represent count measurement reproducibility between single and dual acquisitions. This means that the lower sub-window for I-123, which was narrow and near to the photopeak, caused overestimation of scatter; however, the FiveEW method reduced this scatter overestimation and thereby improved the linearity and reproducibility of activity measurement. On the other hand, the results of the simulation and experiments show that the effect of using DR for Tc-99m measurement was limited, which suggests that the effect of using DR depends on the lower window setting.

The FiveEW method corrected for scatter and contamination while preserving the linearity of activity measurement (Fig. 7, Table 5). Although the accuracy of Tc-99m measurement for dual acquisition was less than that of I-123 measurement (Fig. 7), the FiveEW method had higher quantitative accuracy for simultaneous dual acquisition.

In this study, we evaluated the performance of simultaneous Tc-99m and I-123 dual-radionuclide imaging. The photopeak gap between Tc-99m and I-123 is the narrowest among all the commonly used radiopharmaceutical pairs. When imaging other radionuclide pairs with a wider photopeak gap, sharing an upper window for lower energy photopeaks and a lower window for higher energy photopeaks is unnecessary. Eq. 4 was solved analytically because the TEW algorithm is very simple and linear. If the scatter-correction method is not simple, Eq. 4 can be solved using an iterative method, such as

10 $$ {P}_L^{k+1}=r\cdot {P}_M=r\cdot \mathrm{S}\mathrm{C}\left({E}_L-{P}_L^k,{E}_M,{E}_U\right) $$

where *k* is the iteration index, ${P}_L^k$ is the primary count map of the *k*th iteration, and SC is an energy-window-based scatter correction term. Further investigation for optimizing the SC algorithm such as by investigating a non-linear scatter spectrum model and identifying the optimum energy window setting should lead to more accurate simultaneous dual-radionuclide scanning.

There are various radiopharmaceuticals for cerebral perfusion, benzodiazepine receptor, dopamine receptor/transporter, serotonin transporter, and other functional imaging [30, 31]. In addition, some amyloid-β biomarkers are under development [32, 33]. We believe that simultaneous acquisition for these molecular functions and perfusion will be important. Since temporal changes in these functions may be seen in dementia, epilepsy, depression, traumatic injury, cerebrovascular disease, and neuroinflammation, clinical scientists would like to assess various functions as well as molecular parameters at the same time rather than a different time. SPECT has the potential to obtain multiple parameters using two or more different radiopharmaceuticals at the same time using different energy windows. While dual tracer protocols to perform back-to-back acquisition with correction using differences in kinetics, spatial distribution and/or decay time have been developed [34, 35], simultaneous multi-tracer imaging with positron emission tomography (PET) is still challenging. Hybrid PET-magnetic resonance imaging (PET-MRI) has a potential to obtain multiple biological processes with simultaneous acquisition [36, 37]. Although spatial resolution and sensitivity of SPECT are worse than PET-MRI, SPECT will play a significant role in diagnoses of cranial nerve diseases because of the lower cost and the availability of radiopharmaceuticals.

While simultaneous dual-radionuclide imaging has been demonstrated in various studies [4–13], simultaneous Tc-99m and I-123 imaging is not commonly used in daily clinical situations. One reason is that additional effort and time are needed to correct for scatter and crosstalk. Another reason is that the quantitative accuracy has not been sufficiently confirmed for clinical use, especially for the Tc-99m–I-123 pair for brain imaging. The results of our experiments demonstrated that the quantitative accuracy and image quality of our CdTe solid-state SPECT prototype system and correction method are good enough to enable simultaneous Tc-99m and I-123 clinical imaging. Moreover, the method used for scatter and crosstalk correction is simple and fast enough for use in daily clinical scanning. The combination of CdTe-SPECT and the correction method will permit simultaneous quantitative Tc-99m and I-123 study. The clinical usefulness of simultaneous multi-functional imaging using multi-radionuclide SPECT scan or PET-MRI is still under research; however, we believe that the potential of simultaneous multi-functional imaging will contribute to the diagnosis and identification of the mechanisms of diseases.

There are three limitations to this study. One is ignoring the higher photopeak of I-123 (529 keV) for detector response correction in the FiveEW method. Another is that the scatter-corrected data are no longer Poisson distributed, so OSEM is not entirely appropriate. The other is the limited evaluation of human imaging. To overcome these limitations, we need to evaluate the partial volume effect, attenuation correction accuracy, and higher I-123 photopeak effect and conduct further clinical evaluation.

## Conclusions

We have developed a prototype CdTe brain imaging SPECT system and a scatter and crosstalk correction method using five energy windows for simultaneous Tc-99m and I-123 brain imaging. Our prototype SPECT system provides quantitative and high-quality brain perfusion images and cerebral blood volume images simultaneously without position mismatch or temporal difference. The solid-state SPECT system will permit quantitative dual-radionuclide brain studies to become clinically available and contribute to research and diagnosis of cranial nerve diseases such as dementia.

## Abbreviations

CdTe, cadmium telluride; CBF, cerebral blood flow; CBV, cerebral blood volume; DRC, detector response correction; FiveEW, Five energy window; FiveEW-woDR, FiveEW without detector response correction; HSA-D, human serum albumin diethylene triamine pentaacetic acid; IMP, *N*-isopropyl-4-iodoamphetamine hydrochloride; NC, no correction; OSEM, ordered subset estimation maximization; PD, percentage difference; PET, positron emission tomography; PET-MRI, hybrid PET-magnetic resonance imaging; PSF, point spread function; ROI, region of interest; RS, residual scatter; SD, standard deviation; SPECT, single-photon emission computed tomography; TEW, triple-energy window; TEWDR, TEW with DRC; 4-PMC, 4-pixel matched collimators
